# Different dietary starch sources alter the carcass traits, meat quality, and the profile of muscle amino acid and fatty acid in finishing pigs

**DOI:** 10.1186/s40104-020-00484-9

**Published:** 2020-08-07

**Authors:** Miao Yu, Zhenming Li, Ting Rong, Gang Wang, Zhichang Liu, Weidong Chen, Jiazhou Li, Jianhao Li, Xianyong Ma

**Affiliations:** Institute of Animal Science, Guangdong Academy of Agricultural Sciences; State Key Laboratory of Livestock and Poultry Breeding; Key Laboratory of Animal Nutrition and Feed Science in South China, Ministry of Agriculture and Rural Affairs; Guangdong Provincial Key Laboratory of Animal Breeding and Nutrition; Guangdong Engineering Technology Research Center of animal Meat quality and Safety Control and Evaluation; Maoming Branch, Guangdong Laboratory for Lingnan Modern Agriculture, Guangzhou, 510640 Guangdong China

**Keywords:** Amino acid profile, Fatty acid composition, Finishing pigs, Lipid metabolism, Meat quality, Starch source

## Abstract

**Background:**

With increasing health awareness among consumers, the demand for healthier, tastier, higher quality and nutritional value pork is increasing. It has been shown that different dietary starch sources can alter the carcass traits and meat quality. However, research on the effects of different starch sources with clear different amylose/amylopectin ratio on the amino acid and fatty acid composition in *Longissimus thoracis* (*L*. *thoracis*) muscle of pigs is limited. This study aimed to investigate the effects of different dietary starch sources on carcass traits, meat quality, muscle amino acid and fatty acid composition, and the mRNA expression levels of genes involved in lipid metabolism and muscle fiber characteristics in finishing pigs. A total of 72 Duroc × Landrace × Large White barrows were randomly allocated to 3 different dietary treatment groups with 8 replicate pens/group and 3 pigs per pen. Tapioca starch (TS), corn starch (CS), and pea starch (PS), with amylose/amylopectin ratio of 0.11, 0.25, and 0.44, respectively, were used as their dietary starch sources for 40 days.

**Results:**

Results showed that the PS diet significantly increased (*P* < 0.05) the final body weight, average daily gain, loin-eye area, and fat-free lean index compared with the TS diet, but significantly decreased (*P* < 0.05) the feed to gain ratio and backfat thickness. Compared with the TS diet, PS diet also increased (*P* < 0.05) the pH_45 min_, marbling scores, the content of intramuscular fat and inosine monophosphate in the *L*. *thoracis*, and decreased (*P* < 0.05) the drip loss and shear force. In addition, compared with the TS diet, PS diet increased (*P* < 0.05) the proportions of flavor amino acids, DHA, EPA, and n-3 polyunsaturated fatty acid (PUFA) in the *L*. *thoracis* compared with TS diet, but decreased (*P* < 0.05) the ratio of n-6/n-3 PUFA. Furthermore, compared with the TS diet, PS diet also upregulated (*P* < 0.05) the lipogenic genes (*FAS*, *LPL*, *SCD*, *ACCα*) and myosin heavy-chain (*MyHC*)-*IIa* mRNA expression levels compared with the TS diet, but downregulated (*P* < 0.05) the *CPT1B* and *MyHC-IIb* mRNA levels.

**Conclusions:**

In conclusion, these results provided compelling evidence that the different dietary starch source altered the carcass traits, meat flavor and quality in finishing pigs, and consumption of a diet with higher amylose/amylopectin ratio results in the production of a healthy, higher quality, and nutritional value pork.

## Background

Pork is one of the most widely consumed meats in the world. The genetic selection of commercial pig lines for higher growth rate and lean percentage may in part lead to the decrease in the quality and nutritional value of pork. However, the demand for safer, healthier, tastier, higher quality and nutritional value pork by consumers is increasing [1]. Intramuscular fat (IMF) plays an important role in various aspects of meat quality and is also critical to its nutritional value [2]. Higher content of IMF can increase the meat quality as it contributes to pork tenderness, flavor, and juiciness [3]. In addition, the fatty acid profiles of muscle also play a key role in meat quality. Polyunsaturated fatty acid (PUFA), especially the n-3 fatty acids, are considered as functional ingredients to prevent cardiovascular disease in humans [4]. n-3 PUFA deficiency and excessive content of n-6 PUFA have already been linked to the development of insulin resistance and metabolic disorders [5]. Additionally, the content and composition of free amino acid in the muscle, particularly the flavor amino acids (FAAs), such as glycine, alanine, aspartate, glutamate, phenylalanine, and tyrosine, also play an important role in determining the nutritional value of meat and directly affects its taste properties [6, 7]. Feeding strategies can change several aspects of meat quality. Accordingly, though the use of nutritional approaches, meat producers should be able to consistently produce and supply high quality pork that contains higher level of IMF and FAAs, as well as a balanced fatty acid composition.

Starch is composed of two types of molecules, amylose and amylopectin, and is the main dietary source of carbohydrates and energy for humans and monogastric animals [8, 9]. Due to their molecular configuration and structure, amylose is resistant to digestion in the small intestine, while amylopectin is easily digestible and may lead to a rapid increase in postprandial glucose and insulin levels in blood [10]. Thus, diets with different amylose/amylopectin ratios may result in different effects on glucose flux and insulin metabolism in the host. Blood glucose and insulin levels can influence lipid metabolism, glycogen production, and protein turnover in the muscle, thus further changing the meat quality [9, 11]. Indeed, accumulating evidence also indicates that diets containing starch with different amylose/amylopectin ratios distinctly alter the growth performance, as well as carcass traits and pork quality, and these changes may be associated with the alteration of lipid metabolism and muscle fiber characteristics [9, 12, 13]. As mentioned above, amino acids and fatty acids composition in the muscle are the main factors in the nutritional and health values of pork. Doti et al. [12] have found that dietary starch source changed the fatty acid profile of *Longissimus thoracis* (*L. thoracis*) muscle in growing pigs given diets based on barley, barley/broken rice, barley/maize, and barley/peas. To the best of our knowledge, to date, it‘s still relatively limited studies on the effects of diets, containing different starch sources with clearly different amylose/amylopectin ratio, on the amino acid profile and fatty acid composition of pork. Thus, further research on this topic is needed.

In the current study, we hypothesized that dietary starches with higher amylose content can improve the pork quality by increasing the contents of IMF and FAAs, and changing the fatty acid composition in *L. thoracis* muscle of finishing pigs, and these changes may be associated with the expression levels of genes involved in lipid metabolism and muscle fiber composition. Therefore, this study aimed to investigate the effects of different starch sources with clearly different amylose/amylopectin ratio on the carcass traits, meat quality, muscle amino acid and fatty acid composition, and the mRNA expression levels of genes involved in lipid metabolism and muscle fiber characteristics of the *L. thoracis* in finishing pigs.

## Material and methods

### Animals, experimental design, and diets

The present study is a part of a series of studies designed to determine whether diets with different starch sources have different effects on the carcass traits, meat quality, and the profile of muscle amino acids and fatty acid of finishing pigs, and a detailed description of animals, experimental design, and diets has been provided in our previous study [14]. Briefly, 72 Duroc × Landrace × Large White growing barrows, with an average body weight (BW) of 77 ± 0.52 kg, were randomly allocated to 3 different dietary treatment groups. Each treatment group consisted of 8 pens (replicates), with 3 pigs per pen. The tapioca starch (TS) group, corn starch (CS) group, and pea starch (PS) group were used as the dietary starch sources. The starch and the ratio of amylose to amylopectin of the three diets were determined using a Total Starch Kit and Amylose/Amylopectin Kit (Megazyme International Ltd., Wicklow, Ireland), respectively. All experimental diets were formulated to contain similar level of crude protein (CP) and metabolizable energy (ME) and to meet or exceed the nutrient recommendations of the National Research Council (NRC) [15] (Table 1). The diets and water were provided ad libitum throughout the 40-day feeding experiment. The feed intake per pen was measured every day to calculate the average daily feed intake (ADFI). The initial and final BW were recorded to calculate average daily gain (ADG) and the ratio of average daily feed intake to average daily gain (F/G) per pen.

**Table 1:** Feed ingredient and nutrient composition of experimental diets (%, as-fed basis)

Items	Diet^1^		
	TS	CS	PS
Ingredient, %			
Tapioca starch	59.00		
Corn starch		59.00	
Pea starch			59.00
Soybean meal	27.00	27.00	27.00
Corn gluten meal	3.40	3.40	3.40
Wheat bran	3.36	3.36	3.36
Soybean oil	3.25	3.25	3.25
*L*-Lysine·HCl (98%)	0.30	0.30	0.30
*DL*-Methionine	0.13	0.13	0.13
*L*-Threonine	0.06	0.06	0.06
Dicalcium phosphate	1.50	1.50	1.50
Limestone	0.30	0.30	0.30
Choline chloride (50%)	0.40	0.40	0.40
Salt	0.30	0.30	0.30
Vitamin and mineral premix^2^	1.00	1.00	1.00
Total	100.00	100.00	100.00
Calculated content			
ME^3^, MJ/kg	13.81	13.81	13.81
Standardized ileal digestible amino acids^4^, %			
Lysine	0.88	0.88	0.88
Methionine + Cysteine	0.49	0.49	0.49
Threonine	0.50	0.50	0.50
Tryptophan	0.15	0.15	0.15
Analyzed nutrient composition			
Dry matter^5^, %	88.48	88.86	88.45
Crude protein^5^, %	14.54	14.55	14.55
Crude fat^5^, %	1.26	1.25	1.25
Crude ash^5^, %	4.09	4.09	4.11
Total starch, % DM	52.25	52.26	52.25
Amylose/amylopectin	0.11	0.25	0.44

^1^TS: tapioca starch; CS: corn starch; PS: pea starch

^2^Provided per kilogram of complete diet: vitamin A, 15,000 IU; vitamin D_3_, 3,000 IU; vitamin E, 150 mg; vitamin K_3_, 3 mg; vitamin B_1_, 3 mg; vitamin B_2_, 6 mg; vitamin B_6_, 5 mg; vitamin B_12_, 0.03 mg; niacin, 45 mg; vitamin C, 250 mg; calcium pantothenate, 9 mg; folic acid, 1 mg; biotin, 0.3 mg; choline chloride, 500 mg; Fe (FeSO_4_·H_2_O), 170 mg; Cu (CuSO_4_·5H_2_O), 150 mg; I (KI), 0.90 mg; Se (Na_2_SeO_3_),0.2 mg; Zn (ZnSO_4_·H_2_O), 150 mg; Mg (MgO), 68 mg; Mn (MnSO_4_·H_2_O), 80 mg; Co (CoCl_2_), 0.3 mg

^3^ME = metabolized energy

^4^Values were estimated based on database of NRC [15]

^5^Analytical results obtained according to AOAC [16]

### Sample collection and preparation

After 40 days of treatment, 24 pigs (8 pigs/treatment group, 1 per pen with medium BW) were selected. After fasting for approximately 12 h, pigs were slaughtered by exsanguination after euthanasia by electrical stunning. After dehairing, peeling, eviscerating, and then splitting in half along the midline, about 5 g of *L. thoracis* were immediately and aseptically excised from the right side of the carcass and flash frozen in liquid nitrogen, and stored at − 80 °C for subsequent gene expression analysis.

Samples (about 100 g) of *L. thoracis* between the 9^th^ and 10^th^ ribs of the left side were collected and stored at − 80 °C for subsequent IMF, inosine monophosphate (IMP), amino acid profile, and fatty acid profile analysis. The *L. thoracis* from the left side of each carcass between the 10^th^ and 13^th^ ribs were sampled for analysis of meat quality.

### Carcass characteristics and meat quality measurements

Within 30 min of slaughter, the hot carcass weight (HCW) was recorded to calculate the dressing percentage. The left side was cut between the 10^th^ and 11^th^ ribs to determine the loin-eye area (LEA) by using a planimeter and the average of three measurements was used as LEA for each carcass. The average backfat thickness was defined as the average thickness of the 1^st^ rib, lumbar, and last rib. The subjective *L. thoracis* marbling score was determined using the guidelines of the National Pork Producers Council (NPPC, Des Moines, IA, USA) [17]. The fat-free lean index (FFLI) of the carcass was calculated by using the equations of the NPPC guidelines.

The *L. thoracis* pH values at 45 min (pH_45 min_) and 24 h (pH_24 h_) postmortem were measured at 3 locations on the 10^th^ rib interface using an HI 9024C hand-held pH meter (HANNA Instruments, Woonsocket, RI, USA), which was calibrated at the beginning of each measurement day using pH 4.6 and 7.0 buffers equilibrated at 35 °C. Objective color measurements (L*, a*, and b*) were performed at 45 min and 24 h after slaughter using a Minolta CR-400 Chroma meter (Konica Minolta Sensing Inc., Osaka, Japan) calibrated against a standard white plate (8 mm diameter aperture, d/0 illumination system), and the results were expressed as the mean of 3 random readings on the surface of each sample. Drip loss was determined as described by Honikel [18]. Briefly, about 3.0 cm length cube of the 10^th^-rib *L. thoracis* (about 55 g) was manually trimmed and weighed at 45 min postmortem, and suspended by a fishhook in an inflated plastic bag, sealed and stored for 24 h at 4 °C, after which the sample was removed from the fishhook, blotted dry on filter paper, and reweighted. Drip loss was calculated as follows: drip loss (%) = [(initial weight−final weight)/initial weight] × 100%. To measure the shear force, at 24 h postmortem, samples of *L. thoracis* (about 150 g) were placed in a plastic bag, and cooked to an internal temperature of 70 °C in an 80 °C thermostatic water-bath. Then, the cooked samples were cooled to room temperature in running water. Thereafter, the cooked samples were cut to 1 cm × 1 cm × 3 cm sheared parallel to the muscle fiber direction as described by Yu et al. [19]. The shear force (N) of the sample cores was measured by using a C-LT3B digital-display muscle tenderness determination device (Tenovo, Harbin, China) with a load cell of 15 kg and a 200-mm/min crosshead speed.

### Diet and muscle chemical analysis

Dry matter (DM), crude fat, and crude protein (CP) of the experimental diet were determined according to the method of the Association of Official Analytical Chemists (AOAC; Rockville, MD, USA) [16]. Samples of *L. thoracis* were measured for DM and IMF by the methods of the AOAC [16]. The concentration of IMP in the *L. thoracis* samples was determined by high performance liquid chromatography (HPLC) analysis according to Li et al. [20]. To determine the free amino acid profile in the *L. thoracis*, about 0.2 g of freeze-dried muscle samples was homogenized in 1 mL of 0.02 mmol/L HCl. The mixture was vortexed for 15 min and then centrifuged at 13,000×*g* for 15 min at 4 °C. An aliquot of 0.5 mL of the clear supernatant liquid was mixed with 0.5 mL 10% (wt/vol) sodium sulfosalicylate. After centrifuging at 13,000×*g* for 15 min at 4 °C, the supernatant was filtered through a 0.22-μm syringe filter and then analyzed on an ion-exchange AA analyzer (L8900, Hitachi, Tokyo, Japan).

For the analysis of the fatty acid composition of the *L. thoracis*, the samples freeze-dried were extracted total lipids using the chloroform-methanol (1:1, v/v) procedure according to Folch et al. [21]. Fatty acid methyl esters of *L. thoracis* were measured using KOH-methanol as the reagent according to our previous study [19]. Fatty acid methyl esters were separated and analyzed using on Agilent 7890B gas chromatographer (GC) system with a flame ionization detector (Agilent Technologies Inc., Santa Clara, CA, USA), using a method described in Li et al. [20]. The individual fatty acids were identified by the retention times of the peak area compared with those of known standards (Sigma Chemical Co., St. Louis, MO, USA), and expressed as a percentage of total fatty acids.

### Total RNA extraction and real-time quantitative PCR

Total RNA was extracted from the frozen *L. thoracis* samples using the TRizol reagent (Takara Biotechnology, Dalian, China) according to the manufacturer’s instructions. The RNA quality and concentration of every sample were measured using a Nanodrop-1000 spectrophotometer (Thermo Fisher Scientific Inc., Waltham, MA, USA), and the ratio (OD_260_: OD_280_) ranged from 1.8 to 2.0. Thereafter, 1 μg of total RNA was used to produce cDNA using a Synthesis Kit (Takara Biotechnology) according to the manufacturer’s instructions. Real-Time PCR of the lipid metabolism and muscle fiber types genes and *GAPDH* detected on a CFX96 Real-time PCR Detection System (Bio-Rad Laboratories, Hercules, CA, USA) using the TB Green™ Premix Ex Taq™ (Takara Biotechnology) in a total volume of 20 μL and analyzed in triplicate. The specific primers sequences used in this study are listed in Table S1 in the supplemental material. The protocols for all genes included a denaturation program (3 min at 95 °C), amplification and quantification program repeated for 40 cycles (5 s at 95 °C, 30 s at 60 °C), followed by the melting curve program at 65–95 °C with a heating rate of 0.1 °C/s. The relative quantification of gene amplification by Real-Time PCR was determined using the value of threshold cycle (Ct). To normalize the mRNA expression levels of each target gene, *GAPDH* was used as the internal control, and the relative expression levels were calculated using the 2^−(ΔΔCt)^ method [22].

### Statistical analysis

All experimental data in this study were analyzed by the IBM SPSS statistics V20.0.0 software package (IBM Corp., Armonk, NY, USA). The growth performance was evaluated using pen as the experimental unit, while other experimental data were evaluated using individual pigs as the experimental unit. The one-way analysis of variance (ANOVA) followed by a Tukey’s *post-hoc* test was used to analyze all experimental data. The effects of different dietary starch sources that containing different amylose/amylopectin ratio was also tested by orthogonal polynomial contrasts (linear and quadratic). Data were expressed as the mean ± SEM. Differences were identified as significant at *P* ≤ 0.05.

## Results

### Growth performance and carcass traits

The growth performance and carcass traits of finishing pigs fed diets with different starch sources are presented in Table 2. The finial BW, ADG, and FFIL showed a linear and quadratic increased (*P* < 0.05) in response to increasing amylose/amylopectin ratio with a maximum observed for the PS group. Quadratic (*P* < 0.05) increased response was observed for the LEA among the three treatments, with a maximum observed for the PS group. The PS diet significantly decreased (*P* < 0.05) the average backfat thickness compared with the TS group. On the other hand, the F:G was linearly and quadratically improved (*P* < 0.05) in response to increasing amylose/amylopectin ratio with a minimum observed for the PS group. However, the different dietary treatments had no effect (*P* > 0.05) on the ADFI, HCW, and carcass yield.

**Table 2:** Effects of different dietary starch sources on the growth performance and carcass traits of finishing pigs

Items	Treatment			*P*-value^c^		
	TS	CS	PS	ANOVA	Linear	Quadratic
Initial BW, kg	77.21 ± 0.26	77.19 ± 0.23	77.25 ± 0.23	0.980	0.901	0.983
Final BW, kg	109.52 ± 0.91^b^	112.09 ± 1.74^ab^	115.88 ± 1.21^a^	0.011	0.002	0.010
ADG, kg/d	0.85 ± 0.01^b^	0.89 ± 0.04^ab^	0.97 ± 0.03^a^	0.035	0.010	0.034
ADFI, kg/d	2.83 ± 0.07	2.85 ± 0.05	2.89 ± 0.05	0.741	0.438	0.741
F:G	3.34 ± 0.09^a^	3.23 ± 0.11^ab^	3.01 ± 0.04^b^	0.038	0.011	0.038
Hot carcass weight, kg	82.98 ± 1.70	84.12 ± 1.68	85.02 ± 1.52	0.681	0.376	0.681
Carcass yield, %	74.57 ± 0.97	74.79 ± 0.54	75.89 ± 0.54	0.390	0.196	0.390
Backfat, cm	2.45 ± 0.12^a^	2.36 ± 0.12^ab^	2.07 ± 0.04^b^	0.047	0.104	0.056
Loin-eye area, cm	47.26 ± 0.59^b^	50.36 ± 1.24^ab^	51.15 ± 1.59^a^	0.008	0.031	0.075
FFLI, %	51.14 ± 1.00^b^	53.24 ± 0.57^ab^	54.15 ± 0.68^a^	0.034	0.011	0.034

Values are mean ± SEM, *n* = 8

^a,b^Means in the same row with different superscripts differ (*P* < 0.05)

Abbreviations: TS, tapioca starch; CS, corn starch; PS, pea starch; BW, body weight; ADG, average daily gain; ADFI, average daily feed intake; F:G, feed to gain ratio; FFLI, fat-free lean index

^c^The *P* values indicate the effects of different dietary starch sources with different amylose/amylopectin ratios by one-way ANOVA and polynomial contrasts- linear and quadratic analyses, respectively

### Meat quality

As shown in Table 3, different dietary starch sources changed the meat quality of finishing pigs. The pH_45 min_ values and marbling scores were linearly and quadratically increased (*P* < 0.05) in response to increasing amylose/amylopectin ratio, with a maximum observed for the PS group. On the other hand, the drip loss and shear force were linearly and quadratically decreased (*P* < 0.05) in response to increasing amylose/amylopectin ratio, with a minimum observed for the PS group. However, their pH_24 h_ values, and 45 min and 24 h- L* values, a* value, and b* value was not affected (*P* > 0.05) by dietary treatments.

**Table 3:** Effects of different dietary starch sources on the meat quality of finishing pigs

Items	Treatment			*P*-value^c^		
	TS	CS	PS	ANOVA	Linear	Quadratic
pH_45 min_	6.13 ± 0.07^b^	6.28 ± 0.04^ab^	6.31 ± 0.03^a^	0.042	0.019	0.042
pH_24 h_	5.50 ± 0.03	5.53 ± 0.02	5.54 ± 0.04	0.967	0.794	0.967
L* _45 min_	43.35 ± 0.71	44.97 ± 0.54	44.00 ± 0.51	0.181	0.474	0.181
a* _45 min_	16.68 ± 0.72	17.09 ± 0.52	18.28 ± 0.48	0.206	0.096	0.206
b* _45 min_	2.21 ± 0.34	2.47 ± 0.32	2.11 ± 0.28	0.698	0.814	0.698
L* _24 h_	55.36 ± 0.60	56.25 ± 0.79	55.55 ± 0.76	0.657	0.854	0.657
a* _24 h_	16.46 ± 0.19	16.04 ± 0.32	16.57 ± 0.29	0.361	0.764	0.361
b* _24 h_	2.66 ± 0.24	2.72 ± 0.29	3.01 ± 0.29	0.529	0.289	0.529
Marbling scores	2.44 ± 0.13^b^	2.70 ± 0.16^ab^	3.04 ± 0.15^a^	0.028	0.007	0.028
Drip loss, %	3.22 ± 0.16^a^	2.84 ± 0.13^ab^	2.73 ± 0.11^b^	0.043	0.016	0.043
Shear force, *N*	38.39 ± 1.96^a^	34.63 ± 2.71^ab^	31.09 ± 1.02^b^	0.049	0.016	0.050

Values are mean ± SEM, *n* = 8

^a,b^Means in the same row with different superscripts differ (*P* < 0.05)

Abbreviations: TS, tapioca starch; CS, corn starch; PS, pea starch; L*, lightness; a*, redness; b*, yellowness

^c^The *P* values indicate the effects of different dietary starch sources with different amylose/amylopectin ratios by one-way ANOVA and polynomial contrasts- linear and quadratic analyses, respectively

### *Longissimus thoracis* muscle chemical composition

As shown in Table 4, the content of IMF and IMP in the *L. thoracis* of finishing pigs were linearly and quadratically increased (*P* < 0.05) in response to increasing amylose/amylopectin ratio, with a maximum observed for the PS group. However, different dietary treatment did not affect (*P* > 0.05) the DM content.

**Table 4:** Effects of different dietary starch sources on the *longissimus thoracis* muscle chemical composition of finishing pigs (as-fresh basis)

Items	Treatment			*P*-value^c^		
	TS	CS	PS	ANOVA	Linear	Quadratic
DM, %	27.25 ± 0.36	26.90 ± 0.25	27.16 ± 0.32	0.717	0.832	0.717
IMF, %	3.81 ± 0.17^b^	4.22 ± 0 30^ab^	4.65 ± 0.13^a^	0.035	0.004	0.019
IMP, mg/g	1.72 ± 0.05^b^	1.78 ± 0.04^ab^	1.89 ± 0.04^a^	0.045	0.007	0.027

Values are mean ± SEM, *n* = 8

^a,b^Means in the same row with different superscripts differ (*P* < 0.05)

Abbreviations: TS, tapioca starch; CS, corn starch; PS, pea starch; DM, dry matter; IMF, intramuscular fat; IMP, inosine monophosphate

^c^The *P* values indicate the effects of different dietary starch sources with different amylose/amylopectin ratios by one-way ANOVA and polynomial contrasts- linear and quadratic analyses, respectively

### Free amino acid profiles in *longissimus thoracis* muscle

The free amino acid profiles of the *L. thoracis* are shown in Table 5. The concentration of aspartate, glycine, and total FAAs in the *L. thoracis* of finishing pigs showed a linear and quadratic increased (*P* < 0.05) in response to increasing amylose/amylopectin ratio, with a maximum observed for the PS group. In addition, the alanine concentration also showed a quadratic increased (*P* < 0.05) in response to increasing amylose/amylopectin ratio, with a maximum observed for the PS group. However, different dietary treatments did not affect (*P* > 0.05) other amino acids and total amino acid concentrations.

**Table 5:** Effects of different dietary starch sources on the free amino acid profile of the *longissimus thoracis* muscle of finishing pigs (mg/100 g)

Items	Treatment			*P*-value^c^		
	TS	CS	PS	ANOVA	Linear	Quadratic
Aspartate	3.24 ± 0.29^b^	4.14 ± 0.49^b^	6.90 ± 0.91^a^	0.001	< 0.001	0.001
Serine	35.85 ± 2.41	34.94 ± 1.05	36.78 ± 1.92	0.788	0.724	0.788
Glutamate	84.43 ± 4.68	88.30 ± 5.84	76.04 ± 3.66	0.207	0.239	0.207
Glycine	134.26 ± 2.22^b^	141.37 ± 4.85^b^	158.28 ± 5.53^a^	0.003	0.001	0.003
Alanine	340.53 ± 6.36^b^	350.86 ± 5.63^ab^	361.49 ± 6.68^a^	0.048	0.024	0.082
Tyrosine	41.47 ± 1.91	45.65 ± 1.72	45.28 ± 2.93	0.366	0.242	0.366
Cysteine	4.03 ± 0.15	4.03 ± 0.23	4.19 ± 0.19	0.797	0.553	0.797
Proline	40.53 ± 1.47	45.20 ± 3.89	39.64 ± 1.57	0.279	0.814	0.279
Asparagine	12.06 ± 0.48	12.09 ± 0.55	12.47 ± 0.68	0.857	0.612	0.857
Glutamine	155.29 ± 11.27	143.67 ± 11.24	166.84 ± 15.05	0.446	0.527	0.446
Valine	56.40 ± 2.46	55.22 ± 3.02	54.90 ± 2.82	0.922	0.700	0.922
Threonine	37.41 ± 1.90	38.41 ± 2.44	37.09 ± 1.81	0.895	0.913	0.895
Methionine	25.22 ± 1.34	25.03 ± 0.89	23.78 ± 2.07	0.768	0.499	0.768
Isoleucine	31.00 ± 1.33	31.33 ± 1.74	30.95 ± 2.12	0.986	0.984	0.986
Leucine	72.97 ± 4.37	70.20 ± 2.13	70.24 ± 3.99	0.827	0.592	0.827
Phenylalanine	44.51 ± 2.06	47.75 ± 1.96	48.80 ± 2.46	0.363	0.168	0.363
Lysine	66.66 ± 4.66	58.48 ± 4.07	70.12 ± 6.85	0.304	0.659	0.304
Histidine	25.13 ± 1.11	26.80 ± 1.67	24.10 ± 1.15	0.370	0.599	0.370
Arginine	15.82 ± 2.51	13.52 ± 1.09	13.97 ± 2.02	0.662	0.488	0.662
Tryptophan	162.12 ± 5.70	159.43 ± 11.77	157.02 ± 11.63	0.938	0.718	0.938
FAA	648.43 ± 6.67^b^	678.07 ± 10.90^ab^	696.77 ± 14.02^a^	0.017	0.004	0.017
TAA	1388.90 ± 19.58	1396.43 ± 15.56	1438.87 ± 22.17	0.165	0.078	0.167

Values are mean ± SEM, *n* = 8

^a,b^Means in the same row with different superscripts differ (*P* < 0.05)

Abbreviations: TS, tapioca starch; CS, corn starch; PS, pea starch; FAA, flavor amino acid; TAA, total amino acid

^c^The *P* values indicate the effects of different dietary starch sources with different amylose/amylopectin ratios by one-way ANOVA and polynomial contrasts- linear and quadratic analyses, respectively

### Fatty acid profiles in *longissimus thoracis* muscle

As shown in Table 6, different dietary starch sources significantly altered the fatty acid profiles in the *L. thoracis* of finishing pigs. The concentration of C12:0, C18:3n-6, C20:3n-3, C20:4n-6, eicosapentaenoic acid (EPA), docosahexaenoic acid (DHA), and the sum of n-3 PUFA were linearly and quadratically increased (*P* < 0.05) in response to increasing amylose/amylopectin ratio, with a maximum observed for the PS group. Quadratic (*P* < 0.05) increased response was observed for the C16:1n-7 and C22:1 concentration among the three treatments, with a maximum observed for the PS and CS groups, respectively. Additionally, the concentration of C20:1, C20:2, and the ratio of n-6/n-3 PUFA were linearly and quadratically decreased (*P* < 0.05) in response to increasing amylose/amylopectin ratio, with a minimum observed for the PS group. However, although the concentration of a number of fatty acids in the *L. thoracis* muscle of finishing pigs were altered by dietary treatments, the sum of saturated fatty acid (SFA), monounsaturated fatty acid (MUFA), PUFA, and the sum of n-6 PUFA were not affect (*P* > 0.05) by different dietary treatments.

**Table 6:** Effects of different dietary starch sources on the fatty acid profile of the *longissimus thoracis* muscle of pigs (% of total fatty acids)

Items	Treatment			*P*-value^e^		
	TS	CS	PS	ANOVA	Linear	Quadratic
C10:0	0.13 ± 0.01	0.14 ± 0.00	0.15 ± 0.00	0.198	0.065	0.198
C12:0	0.11 ± 0.00^b^	0.13 ± 0.00^b^	0.15 ± 0.01^a^	< 0.001	< 0.001	< 0.001
C14:0	1.56 ± 0.02	1.48 ± 0.03	1.58 ± 0.06	0.152	0.747	0.152
C14:1	0.03 ± 0.00	0.02 ± 0.00	0.04 ± 0.00	0.082	0.203	0.082
C15:0	0.03 ± 0.00	0.03 ± 0.00	0.03 ± 0.00	0.463	0.683	0.463
C16:0	24.90 ± 0.23	24.33 ± 0.32	24.35 ± 0.48	0.476	0.698	0.466
C16:1n-7	3.37 ± 0.22^ab^	3.16 ± 0.06^b^	3.88 ± 0.19^a^	0.021	0.065	0.021
C17:0	0.15 ± 0.01	0.15 ± 0.01	0.15 ± 0.01	0.956	0.889	0.956
C18:0	12.92 ± 0.49	13.01 ± 0.34	12.08 ± 0.42	0.256	0.174	0.253
C18:1n-9	42.46 ± 0.64	41.72 ± 0.87	41.80 ± 0.75	0.751	0.198	0.592
C18:2n-6	10.41 ± 0.54	11.55 ± 0.36	10.65 ± 0.42	0.190	0.729	0.190
C18:3n-3	0.55 ± 0.02	0.57 ± 0.01	0.60 ± 0.01	0.207	0.080	0.207
C18:3n-6	0.05 ± 0.00^c^	0.06 ± 0.00^b^	0.07 ± 0.01^a^	< 0.001	< 0.001	< 0.001
C20:0	0.24 ± 0.01	0.23 ± 0.00	0.24 ± 0.01	0.865	0.895	0.865
C20:1	0.81 ± 0.05^a^	0.76 ± 0.01^ab^	0.75 ± 0.01^b^	0.014	0.030	0.014
C20:2	0.47 ± 0.02^a^	0.46 ± 0.01^a^	0.38 ± 0.01^b^	0.003	0.002	0.003
C20:3n-6	0.18 ± 0.01	0.20 ± 0.00	0.20 ± 0.01	0.056	0.051	0.056
C20:3n-3	0.11 ± 0.00^b^	0.11 ± 0.00^b^	0.13 ± 0.00^a^	0.008	< 0.001	< 0.001
C20:4n-6	1.01 ± 0.03^c^	1.42 ± 0.08^b^	1.89 ± 0.06^a^	< 0.001	0.031	0.008
C20:5n-3(EPA)	0.04 ± 0.00^c^	0.08 ± 0.00^b^	0.10 ± 0.00^a^	< 0.001	< 0.001	< 0.001
C22:0	0.03 ± 0.00^b^	0.05 ± 0.00^a^	ND^d^	0.001	0.002	0.002
C22:1	0.03 ± 0.00^b^	0.07 ± 0.00^a^	0.06 ± 0.00^a^	< 0.001	0.060	< 0.001
C22:6n-3 (DHA)	0.12 ± 0.01^b^	0.16 ± 0.01^a^	0.18 ± 0.01^a^	< 0.001	< 0.001	< 0.001
C24:0	0.18 ± 0.01	0.24 ± 0.03	0.24 ± 0.03	0.159	0.072	0.159
SFA	40.27 ± 0.67	39.64 ± 0.65	39.27 ± 0.80	0.606	0.317	0.606
MUFA	46.79 ± 0.79	45.78 ± 0.91	46.62 ± 0.85	0.672	0.890	0.672
PUFA	12.93 ± 0.58	14.60 ± 0.45	14.16 ± 0.46	0.073	0.112	0.074
PUFA n-6	11.64 ± 0.56	13.24 ± 0.42	12.82 ± 0.45	0.183	0.111	0.073
PUFA n-3	0.77 ± 0.03^b^	0.88 ± 0.03^ab^	0.99 ± 0.02^a^	0.004	< 0.001	< 0.001
PUFA n-6/PUFA n-3	15.33 ± 0.94^a^	15.12 ± 0.58^ab^	12.90 ± 0.46^b^	0.020	0.022	0.040

Values are mean ± SEM, *n* = 8

^a,b,c^Means in the same row with different superscripts differ (*P* < 0.05)

SFA- sum of saturated fatty acids; MUFA- sum of monounsaturated fatty acids; PUFA- sum of polyunsaturated fatty acids

^d^ND means not detected

^e^The *P* values indicate the effects of different dietary starch sources with different amylose/amylopectin ratios by one-way ANOVA and polynomial contrasts- linear and quadratic analyses, respectively

### mRNA expression levels of lipid metabolism and myosin heavy-chain-related genes in *longissimus thoracis* muscle

**Fig. 1:**
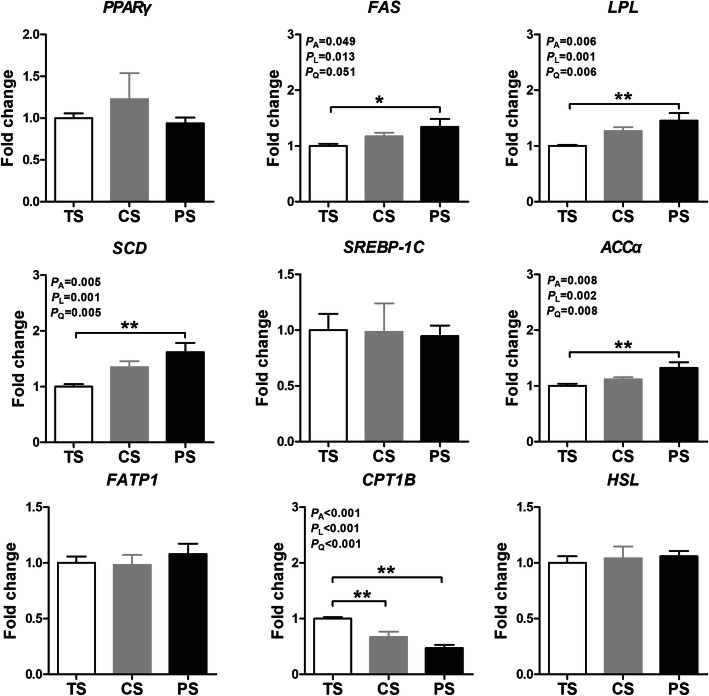
Effects of different dietary starch sources on the relative mRNA expression of genes related to lipid metabolism in *longissimus thoracis* muscle of finishing pigs. The relative mRNA expression levels of peroxisome proliferator-activated receptor γ (*PPARγ*), fatty acid synthase (*FAS*), acetyl CoA carboxylase α (*ACCα*), Sterol regulatory element binding protein-1c (*SREBP-1C*), lipoprotein lipase (*LPL*), carnitine palmitoyl transferase 1B (*CPT1B*), fatty acid transport protein 1 (*FATP1*), and hormone-sensitive lipase (*HSL*) were normalized using β-actin as an internal control. The values are means ± SEM (*n* = 8). The *P* values indicate the effects of different dietary starch sources with different amylose/amylopectin ratios by one-way ANOVA and polynomial contrasts- linear and quadratic analyses, respectively. *P < 0.05, **P < 0.05 (one-way ANOVA with a Tukey's *post-hoc* test). Abbreviations: TS, tapioca starch; CS, corn starch; PS, pea starch

To further investigate the mechanism underlying the regulation of the IMF content by treatment with different dietary starch sources, the mRNA expression levels of key lipid metabolic factors were determined in the *L. thoracis* of finishing pigs. As shown in Fig. 1, the mRNA expression level of lipoprotein lipase *(LPL*), stearoyl-coenzyme A desaturase (*SCD*), and acetyl CoA carboxylase α (*ACCα*) were linearly and quadratically upregulated (*P* < 0.05) in response to increasing amylose/amylopectin ratio, and the fatty acid synthase (*FAS*) expression also quadratically upregulated (*P* < 0.05), with the maximum observed for the PS group. The carnitine palmitoyl transferase 1B (*CPT1B*) mRNA expression level were linearly and quadratically downregulated (*P* < 0.05) in response to increasing amylose/amylopectin ratio, with the minimum observed for the PS group. However, different dietary starch sources did not affect (*P* > 0.05) the mRNA expression level of peroxisome proliferator-activated receptor γ (*PPARγ*), sterol regulatory element binding protein-1c (*SREBP-1C*), fatty acid transport protein 1 (*FATP1*), and hormone-sensitive lipase (*HSL*).

**Fig. 2:**
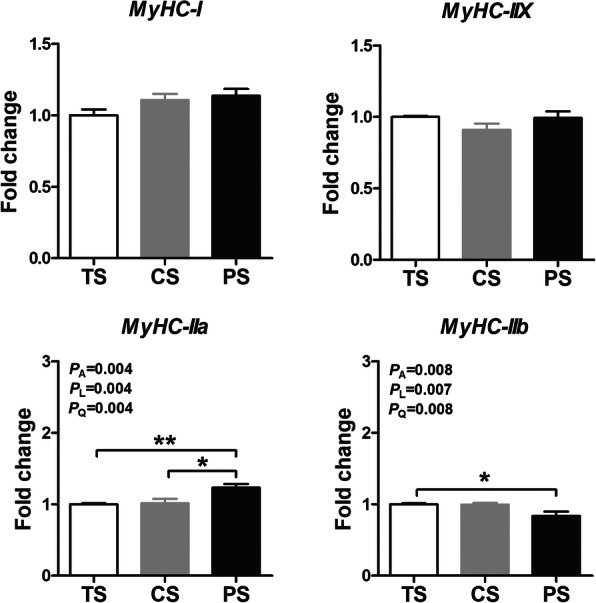
Effects of different dietary starch sources on relative mRNA expression of genes related to myosin heavy-chain (*MyHC*) in *longissimus thoracis* muscle of finishing pigs. The values are means ± SEM (*n* = 8). The *P* values indicate the effects of different dietary starch sources with different amylose/amylopectin ratios by one-way ANOVA and polynomial contrasts- linear and quadratic analyses, respectively. *P < 0.05, **P < 0.01 (one-way ANOVA with a Tukey's *post-hoc* test). Abbreviations: TS, tapioca starch; CS, corn starch; PS, pea starch

As shown in Fig. 2, the mRNA expression level of myosin heavy chain (*MyHC*)-*IIa* were linearly and quadratically upregulated (*P* < 0.05) in response to increasing amylose/amylopectin ratio, with the maximum observed for the PS group. The mRNA expression levels of *MyHC-IIb* were linearly and quadratically downregulated (*P* < 0.05) in response to increasing amylose/amylopectin ratio, with the minimum observed for the PS group. However, the mRNA expression levels of *MyHC-I* and *MyHC-IIx* were not affected (*P* > 0.05) by different dietary treatment. Taken together, these results indicate that pigs fed with PS diet significantly upregulated expression of genes related to the lipogenic potential and muscle fiber composition in the *L. thoracis*.

## Discussion

In recent years, consumers’ demand for pork with high nutritional value and health benefits has been increasing. Starch is the main dietary source of carbohydrates and energy for humans and monogastric animals, and several studies have indicated that different sources of starch may have different physiological effects on pork quality [9, 12, 13]. In the current study, our results indicated that pigs fed with the PS diet (containing a high ratio of amylose) had increased ADG, partly increased carcass traits, IMF, IMP, several FAAs and n-3 PUFA of finishing pigs, and thus the nutritional value and flavor quality of pork were ultimately improved. In addition, these changes were found to be associated with upregulated expression of genes involved in lipid metabolism and muscle fiber composition.

### Different dietary starch sources altered the growth performance and carcass traits

To date, the research findings on the effects of different dietary starch sources on the growth performance of pigs remain controversial. Some studies have found that pigs fed with the non-waxy corn diet (with higher amylose content) did not increase or decreased the growth performance compared with those fed with the waxy corn diet (with higher amylopectin content) [9, 23]. However, in this study, we found that pigs fed with the PS diet (with higher amylose content) increased final BW and ADG compared with those fed with the TS diet (with higher amylopectin content), but had decreased F:G ratio. These results are consistent with the findings of Li et al. [13], which also indicated that pigs fed with the PS diet grew faster and were more feed efficient than those fed with the waxy maize starch diet. Diets with higher amylose/amylopectin ratios can reduce endogenous digestibility in the small intestine and subsequently increase more carbohydrates reaching the large intestine for microbial fermentation to produce short-chain fatty acids (SCFAs). Although the energy utilization of fermentation is less effective than an enzymatic hydrolysis of carbohydrates, a faster starch digestion rate may lead to a sharp but short increment in postprandial blood glucose, which might be more efficient for fat deposition. Instead, a slowly digestible starch could gradually increase the glucose concentration and prolong the release of insulin into the blood, which might favor lean deposition [12]. Tess et al. [24] has demonstrated that the energy cost of adipose tissue deposition was higher than that of muscle tissue, which suggested that lean deposition was more effective for BW gain. Thus, the higher ADG and lower F:G in pigs fed with the PS diet may be due to the higher amylose content of PS, which prolonged the continuous release and absorption of glucose and then cause more lean deposition. However, further studies are needed to elucidate the underlying mechanisms.

In pork production, the excessive deposition of subcutaneous adipose tissue in finishing pigs not only decreases the feed conversion rate, but also affects the consumer acceptance of pork [25]. Notably, this study demonstrated that pigs fed with the PS diet effectively reduced backfat depth and increased LEA and FFLI compared with those fed with the TS diet, which is consistent with Yang et al. [9] and Wang et al. [26], who also found that pigs fed starch with higher amylose tended to decrease the backfat depth and increase the LEA compared with pigs fed starch with higher amylopectin. These results suggested that pigs fed starch with higher amylose content could reduce the subcutaneous fat deposition and improve the carcass characteristics. Blood glucose level may influence the energy metabolism and fat deposition of host. In the current study, we did not determine the glucose release rate of glucose, but as mentioned above, pigs fed the high amylopectin diet would lead to a sharp but short increment in postprandial blood glucose, which in turn favor nutrients partitioning to fat deposition. Thus, the higher amylose content of PS diet may lead to a gradual release and absorption of glucose, which may partly explain the decreased in backfat thickness of pigs. Additionally, the reduced backfat thickness might be associated with the decreased lipogenic rates in pigs fed with higher amylose. Pigs fed starch with lower digestion rate may reduce the activities of lipogenic enzymes and downregulate the mRNA expression levels of lipid metabolism-related genes, and ultimately lead to decreased lipogenesis in adipose tissues [27]. Thus, the decrease of the backfat thickness may be due to alteration of the activities of lipogenic enzymes or the mRNA expression of lipid metabolism-related genes in adipose tissue by the PS diet, but further studies are required to elucidate the molecular mechanisms.

### Different dietary starch sources altered the meat quality, intramuscular fat, fatty acid, and amino acid profiles in the *longissimus thoracis* muscle

Meat quality is the major factor affecting consumer acceptance, as it determines the tenderness, juiciness, and flavor of pork [28]. The current study found that pigs fed with the PS diet significantly increased the pH_45 min_ and marbling scores of *L. thoracis* and decreased drip loss and shear force compared with those fed with TS diet, suggesting a significant role of the PS diet in improving the pork meat quality. A lower pH value can denature myofibrillar proteins and result in less water binding to these proteins [29]. Many studies have concluded that the drip loss and shear force are closely linked with early postmortem pH [30, 31]. Thus, the decrease in drip loss and shear force of *L. thoracis* in pigs fed with PS diet might be induced by the higher pH_45 min_ in this study.

Additionally, the results of this study revealed that pigs fed with the PS diet also increased IMF and IMP concentration in the *L. thoracis* compared with pigs fed with the TS diet. As is widely known, IMF is one of the key indicators of meat quality and can indirectly affect the tenderness, flavor, and juiciness of cooked meat, and the decrease of the IMF content in the *L. thoracis* to below 2.0–2.5% would negatively influence the sensory properties of pork [2]. As a flavor enhancer, the IMP content in meat is considered as very important indicator for judging the meat quality, as it can directly determine the taste of meat after cooking [32]. Thus, the higher IMF and IMP concentration in the *L. thoracis* indicated that pigs fed with the PS diet (with higher amylose content) may result in increased meat tenderness, juiciness, and flavor.

The fatty acid composition of IMF also plays an important role in meat quality and acceptability of meat products as they can determine the nutritional value and oxidative stability of muscle [33]. The n-6 and n-3 PUFA are the important components in food and diet. Food and diet with higher ratios of n-6/n-3 PUFA or n-3 PUFA content may decrease the risk of metabolic syndrome and inflammation [5, 34]. Thus, maintaining a suitable ratio of n-6/n-3 PUFA or containing higher content of n-3 PUFA in the food and diet is beneficial for the health of animals and humans. In the current study, although the n-6/n-3 PUFA ratios are lower than (4–5):1, and the contents of DHA and EPA in the *L. thoracis* are not enough high, pigs fed with the PS diet changed the fatty acid composition, increased the contents of DHA, EPA, and n-3 PUFA, but decreased the ratio of n-6/n-3 PUFA, which indicated that pigs fed with the PS diet (with higher amylose content) may increase the nutritional value of pork in terms of fatty acid nutrition.

To date, there was little published research about the role of different dietary starch sources in regulating fatty acid composition in *L. thoracis* muscle of pigs, and it is difficult to explain the exact mechanism that pigs fed with PS diet increased these n-3 PUFAs. n-3 PUFAs can not be synthesized *de novo* and must be obtained from the diet [35]. Meanwhile, dietary n-3 PUFAs can be bio-hydrogenated by microbes after they enter the gastrointestinal tract [36]. Starch, especially amylose can be fermented by microbes and favor propionate production, which serves as a competitive pathway for H_2_ use in the gut [37]. In our study, the ADFI of pigs did not differ among the three groups, but the intestinal microbe flora and their metabolites were changed, such as the abundance of *Lactobacillus*, *Prevotella*, *Faecalibacterium*, and *Megasphaera* were increased in the PS group, and the propionate concentration was also increased [14, 38]. Thus, the C18:3n-3 is higher in PS group may be due to the PS diet changed the intestinal microbe flora and their metabolites, and then decreased the microbial bio-hydrogenation activity of these fatty acids in the gut, final lead more C18:3n-3, DHA, EPA, and n-3 PUFA absorbed from the gut and deposited in *L thoracis* muscle, but further studies need to elucidate the exact mechanism.

The amino acid profiles in the muscle can determine the quality of the protein. In the current study, we found that the amino acids profiles in the *L. thoracis* was significantly altered by different starch sources diets. Specifically, our results indicated that pigs fed with the PS diet increased the concentration of aspartate, glycine, alanine, and the sum of FAAs in the *L. thoracis*. In meat, these amino acids are generally considered as the precursors of flavoring substances, and can also react with soluble reducing sugars (such as glucose and fructose) to form flavoring substances, and thereby directly affect the freshness of the meat [25, 39]. Thus, the increased levels of aspartate, glycine, alanine, and FAAs by the PS diet in our study may improve the flavor of the pork, making it more favorable to consumers.

So far, the exact mechanism for the change the amino acid profiles of *L thoracis* is unclear. As mentioned above, amylopectin was rapidly digested but amylose was slowly digested, and the higher digesta passage rate of amylopectin could decrease the contact time between feeds and enzymes and may result in incomplete hydrolysis of dietary proteins, finally lead to reduce amino acid absorption [40]. Regmi et al. [41] found that pigs fed with high amylose diets decreased the ileal CP flow, but increased small intestinal CP and amino acids digestion. Xie et al. [42] also found that pigs fed with PS (with higher amylose level) diet upregulated the mRNA expression level of amino acids transporters compared with TS diet (with higher amylopectin level). Thus, the increased amino acids concentrations in the PS group may be due to a higher level of amylose in the PS diet, and then increase small intestinal CP digestion and amino acids absorption, but need further studies to investigate the underlying mechanisms.

### Different dietary starch sources altered the mRNA expression levels of genes involved in lipid metabolism and muscle fiber composition

As is generally known, the marbling of *L. thoracis* is based on the balance between the fat deposition and removal, which is regulated by many intramuscular lipid metabolism genes [43]. In the current study, pigs fed with PS diet upregulated mRNA expression levels of *FAS*, *LPL*, *SCD*, and *ACCα*, and downregulated the expression level of *CPT1B* compared with pigs fed with the TS diet. These findings are in contrast to previous studies [44, 45], in which dietary starch with higher digestion rate upregulated the expression level of lipid metabolism-related genes in weanling pigs, including *FAS* and *ACCα*. These inconsistencies might be due to the duration time of the experiment, age of the pigs, and the source of starch. As stated above, a slowly digestible starch can gradually increase the blood glucose concentration [46, 47]. Glucose can directly influence lipogenic gene expression through the lipid synthesis pathway in a range of tissues. In the current study, although we did not measure the postabsorptive kinetic of glucose, our results o indicated that the PS diet increased the 12 h post-meal blood glucose concentration (unpublished data). Thus, the alteration of lipid metabolism-related genes in the PS group may be due to the increase of the blood glucose concentration by the PS diet, while additional studies are needed to clarify the underlying mechanisms.

The fatty acid availability can directly affect the IMF deposition in the *L. thoracis*. *LPL* is the critical lipid uptake gene and encodes a protein that participates in the process of fatty acid flux into adipocytes clustered along myofiber fasciculi in the muscle, and then provides the appropriate substrate for IMF synthesis [48]. FAS and ACC were the key regulatory enzymes of fatty acid synthesis [49]. FAS is mainly involved in the final step of fatty acid biosynthesis in muscle [50]. ACCα, which is the major form of ACC, is a key regulatory molecule for the *de novo* biosynthesis of long-chain fatty acids (LCFAs) in the muscle [51]. SCD is the first enzyme converting SFA to MUFA and PUFA, and the higher SCD gene expression in the *L. thoracis* was accompanied by higher IMF and PUFA contents [52]. CPT1B is the rate-limiting enzyme in the transport of LCFAs into the mitochondria when LCFAs are destined for oxidation, and this step is the most regulated step in the muscular control of the utilization of LCFAs [53]. Thus, in this study, the higher IMF and n-3 PUFA content of LM in the PS group may result from the upregulation of the mRNA expression level of lipogenic genes (*FAS*, *LPL*, *SCD*, and *ACCα*), and down-regulation of the mRNA expression level of *CPT1B*.

Generally, there are 4 different kinds of fiber types in skeletal muscle, such as slow-oxidative type I, fast-oxidative type IIa, fast-oxidative glycolytic IIx, and fast-glycolytic IIb, respectively [54]. Multiple studies have indicated that muscle fiber types can also influence many aspects of the pork quality [9, 19, 43]. In general, muscle with a higher percentage of type MyHC-IIb fiber, which has greater glycolytic capacity, can lead to a higher value of drip loss but a lower water holding capacity, thus accounting for lower pork quality [55]. However, the increased percentage of MyHC-I and MyHC-IIa fibers in the muscle would be expected to increase the water holding capacity and tenderness [56]. Our study showed that consumption of higher dietary amylose/amylopectin ratio diet (PS diet vs. TS diet) upregulated the mRNA expression of *MyHC-IIa* in the *L. thoracis*, but downregulated the mRNA expression of *MyHC-IIb*, which is consistent with Li et al. [13], who also found that the PS diet increased the levels of *MyHC-I* and *IIa* and decreased the level of *MyHC-IIb* in the *L. thoracis* of finishing pigs. However, Yang et al. [9] and Wang et al. [26] found that consumption of a low dietary amylose/amylopectin ratio diet decreased the levels of *MyHC-I* and *IIx* and increased the level of *MyHC-IIb* in the *L. thoracis* of finishing pigs. According to the results of this study, it is difficult to explain the difference between our results and those obtained by Yang et al. [9] and Wang et al. [26]. However, Gao et al. [56] pointed out that the butyrate generated from low-digestible saccharides or starch was the activator of PGC-1α, which can stimulate the skeletal muscle fiber to convert from fast to slow [57]. In this study, even though we did not measure the *PGC-1α* expression, we found that the PS diet increased the butyrate concentration [14]. Furthermore, Li et al. [13] also found that the mRNA expression level of *PGC-1α* was upregulated by the PS diet. Therefore, in the current study, the increased proportions of *MyHC-IIa* in the PS diet group might be partially related to the higher level of butyrate generated from dietary slowly digestible starch. In general, the transition of fiber types and the alteration of lipid metabolism-related genes expression levels may partly explain why pigs fed with the PS diet (rich in amylose) can improve the pork quality, especially the tenderness.

## Conclusion

In conclusion, the present study demonstrated that feeding pigs with the PS diet (with higher ratio of amylose to amylopectin) increases the ADG, partly increase the carcass traits and the muscle IMF and IMP content, but decreases the F:G. Thus, PS diet also changes the fatty acid and amino acid composition in the *L. thoracis* of finishing pigs. In addition, these changes may be mediated through the upregulation of the expression of genes involved in lipid metabolism and by promoting the transformation of fast-to-slow muscle fiber. This study provides compelling evidence for the consumption of a diet with higher amylose/amylopectin ratio results in the production of a higher quality and nutritional value pork.

## Supplementary information

**Additional file 1 Table S1.** Primers used for host genes in this study.

## Data Availability

The datasets supporting the conclusions of this article are included within the article.

## Abbreviations

ACCα: Acetyl CoA carboxylase α
ADFI: Average daily feed intake
ADG: Average daily gain
AOAC: Association of Official Analytical Chemists
BW: Body weight
CP: Crude protein
CPT1B: Carnitine palmitoyl transferase 1B
CS: Corn starch
DHA: Docosahexaenoic acid
DM: Dry matter
EPA: Eicosapentaenoic acid
FAA: Flavor amino acid
FAS: Fatty acid synthase
FATP1: Fatty acid transport protein 1
F:G: Feed to gain ratio
FFLI: Fat-free lean index
GC: Gas chromatographer
HCW: Hot carcass weight
HPLC: High performance liquid chromatography
HSL: Hormone-sensitive lipase
IMF: Intramuscular fat
IMP: Inosine monophosphate
LEA: Loin-eye area
LCFAs: Long-chain fatty acids
LPL: Lipoprotein lipase
*L. thoracis*: *Longissimus thoracis*
ME: Metabolizable energy
MUFA: Monounsaturated fatty acids
MyHC-I: Myosin heavy-chain I
MyHC-IIa: Myosin heavy-chain IIa
MyHC-IIb: Myosin heavy-chain IIb
MyHC-IIx: Myosin heavy-chain IIx
NPPC: National Pork Producers Council
NRC: National Research Council
PPARγ: Peroxisome proliferator-activated receptor γ
PS: Pea starch
PUFA: Polyunsaturated fatty acids
SFA: Saturated fatty acids
SREBP-1C: Sterol regulatory element binding protein-1c
TAA: Total amino acid
TS: Tapioca starch

## References

[1] Cheng C, Liu Z, Zhou Y, Wei H, Zhang X, Xia M (2017). Effect of oregano essential oil supplementation to a reduced-protein, amino acid-supplemented diet on meat quality, fatty acid composition, and oxidative stability of Longissimus thoracis muscle in growing-finishing pigs. Meat Sci.

[2] Wood J, Enser M, Fisher A, Nute G, Sheard P, Richardson R (2008). Fat deposition, fatty acid composition and meat quality: a review. Meat Sci.

[3] Wood J, Lambe N, Walling G, Whitney H, Jagger S, Fullarton P (2013). Effects of low protein diets on pigs with a lean genotype. 1. Carcass composition measured by dissection and muscle fatty acid composition. Meat Sci.

[4] Simopoulos AP (2001). N−3 fatty acids and human health: defining strategies for public policy. Lipids..

[5] Simopoulos AP (2008). The importance of the omega-6/omega-3 fatty acid ratio in cardiovascular disease and other chronic diseases. Exp Biol Med.

[6] Pereira PMCC, Vicente AFRB (2013). Meat nutritional composition and nutritive role in the human diet. Meat Sci.

[7] Liu Y, Li Y, Feng X, Wang Z, Xia Z (2018). Dietary supplementation with *Clostridium butyricum* modulates serum lipid metabolism, meat quality, and the amino acid and fatty acid composition of Peking ducks. Poultry Sci..

[8] Yin FG, Zhang Z, Huang J, Yin YL (2010). Digestion rate of dietary starch affects systemic circulation of amino acids in weaned pigs. Brit J Nutr..

[9] Yang C, Chen D, Yu B, Huang Z, Mao X, Yu J (2015). Effect of dietary amylose/amylopectin ratio on growth performance, carcass traits, and meat quality in finishing pigs. Meat Sci.

[10] Singh J, Dartois A, Kaur L (2010). Starch digestibility in food matrix: a review. Trends Food Sci Tech.

[11] Drew M, Schafer T, Zijlstra R (2012). Glycemic index of starch affects nitrogen retention in grower pigs. J Anim Sci.

[12] Doti S, Suárez-Belloch J, Latorre M, Guada J, Fondevila M (2014). Effect of dietary starch source on growth performances, digestibility and quality traits of growing pigs. Livest Sci.

[13] Li YJ, Li JL, Zhang L, Gao F, Zhou GH (2017). Effects of dietary starch types on growth performance, meat quality and myofibre type of finishing pigs. Meat Sci.

[14] Yu M, Li Z, Chen W, Rong T, Wang G, Ma X (2019). Microbiome-metabolomics analysis investigating the impacts of dietary starch types on the composition and metabolism of colonic microbiota in finishing pigs. Front Microbiol.

[15] NRC. Nutrient requirements of swine. 11th ed Washington, DC: Natl Acad Press. 2012.

[16] AOAC (2007). Official methods of analysis. 18th ed.

[17] NPPC. National pork producers council. Procedures to Evaluate Market Hogs, third ed National Pork Producers Council, Des Moines, IA. 2000.

[18] Honikel KO (1998). Reference methods for the assessment of physical characteristics of meat. Meat Sci.

[19] Yu M, Li Z, Chen W, Rong T, Wang G, Li J (2019). Use of *Hermetia illucens* larvae as a dietary protein source: effects on growth performance, carcass traits, and meat quality in finishing pigs. Meat Sci.

[20] Li FN, Duan YH, Li YH, Tang YL, Geng MM, Oladele OA (2015). Effects of dietary n-6:n-3 PUFA ratio on fatty acid composition, free amino acid profile and gene expression of transporters in finishing pigs. Brit J Nutr.

[21] Folch J, Lees M, Stanley GS (1957). A simple method for the isolation and purification of total lipides from animal tissues. J Biol Chem.

[22] Yu M, Mu CL, Yang YX, Zhang CJ, Su Y, Huang Z (2017). Increases in circulating amino acids with in-feed antibiotics correlated with gene expression of intestinal amino acid transporters in piglets. Amino Acids.

[23] Camp L, Southern L, Bidner T (2003). Effect of carbohydrate source on growth performance, carcass traits, and meat quality of growing-finishing pigs. J Anim Sci.

[24] Tess M, Dickerson G, Nienaber J, Yen J, Ferrell C (1984). Energy costs of protein and fat deposition in pigs fed ad libitum. J Anim Sci.

[25] Xu X, Chen X, Chen D, Yu B, Yin J, Huang Z (2019). Effects of dietary apple polyphenol supplementation on carcass traits, meat quality, muscle amino acid and fatty acid composition in finishing pigs. Food Funct.

[26] Wang H, Pu J, Chen D, Tian G, Mao X, Yu J (2019). Effects of dietary amylose and amylopectin ratio on growth performance, meat quality, postmortem glycolysis and muscle fibre type transformation of finishing pigs. Arch Anim Nutr.

[27] Martinez-Puig D, Mourot J, Ferchaud-Roucher V, Anguita M, Garcia F, Krempf M (2006). Consumption of resistant starch decreases lipogenesis in adipose tissues but not in muscular tissues of growing pigs. Livest Sci.

[28] Mehta N, Ahlawat S, Sharma D, Dabur R (2015). Novel trends in development of dietary fiber rich meat products-a critical review. J Food Sci Tech.

[29] Hamm R, Bechtel PJ (1986). Functional properties of the myofibrillar system and their measurements. Muscleas food.

[30] Rosenvold K, Andersen HJ (2003). Factors of significance for pork quality-a review. Meat Sci.

[31] Lefaucheur L, Lebret B, Ecolan P, Louveau I, Damon M, Prunier A (2011). Muscle characteristics and meat quality traits are affected by divergent selection on residual feed intake in pigs. J Anim Sci.

[32] Jung S, Bae YS, Kim HJ, Jayasena DD, Lee JH, Park HB (2013). Carnosine, anserine, creatine, and inosine 5′-monophosphate contents in breast and thigh meats from 5 lines of Korean native chicken. Poultry Sci.

[33] Duan YH, Duan YM, Li FN, Li YH, Guo QP, Ji YJ (2016). Effects of supplementation with branched-chain amino acids to low-protein diets on expression of genes related to lipid metabolism in skeletal muscle of growing pigs. Amino Acids.

[34] Robinson LE, Buchholz AC, Mazurak VC (2007). Inflammation, obesity, and fatty acid metabolism: influence of n-3 polyunsaturated fatty acids on factors contributing to metabolic syndrome. Appl Physiol, Nutr Me.

[35] Ma XY, Jiang ZY, Lai CQ (2016). Significance of increasing n-3 PUFA content in pork on human health. Crit Rev Food Sci.

[36] Wahle KWJ, Heys SD, Rotondo D (2004). Conjugated linoleic acids: are they beneficial or detrimental to health?. Progress Lipid Res.

[37] Benchaar C, Pomar C, Chiquette J (2001). Evaluation of dietary strategies to reduce methane production in ruminants: a modelling approach. Can J Anim Sci.

[38] Yu M, Li ZM, Chen WD, Rong T, Wang G, Ma XY (2020). Effects of different starch type diets on main microbes and their metabolites in cecal digesta of finishing pigs. Chine J Anim Nutr.

[39] Zhang W, Xiao S, Samaraweera H, Lee EJ, Ahn DU (2010). Improving functional value of meat products. Meat Sci.

[40] Giusi-Perier A, Fiszlewicz M, Rtrat A (1989). Influence of diet composition on intestinal volatile fatty acid and nutrient absorption in unapesthetized pigs. J Anim Sci.

[41] Regmi PR, Metzler-Zebeli BU, Gänzle MG, van Kempen TA, Matte JJ, Zijlstra RT (2011). Starch with high amylose content and low in vitro digestibility increases intestinal nutrient flow and microbial fermentation and selectively promotes *Bifidobacteria* in pigs. J Nutr.

[42] Xie C, Li YJ, Li JL, Zhang L, Gao F, Zhou GH (2017). Effect of dietary starch types on intestinal digestion and metabolism of finishing pigs. Acta Veterinaria et Zootechnica Sinica.

[43] Li YH, Liu YY, Li FN, Lin Q, Dai QZ, Sun JB (2018). Effects of dietary ramie powder at various levels on carcass traits and meat quality in finishing pigs. Meat Sci.

[44] He J, Chen DW, Yu B (2010). Metabolic and transcriptomic responses of weaned pigs induced by different dietary amylose and amylopectin ratio. PLoS One.

[45] Yin FG, Yin YL, Zhang ZZ, Xie MY, Huang J, Huang RL (2011). Digestion rate of dietary starch affects the systemic circulation of lipid profiles and lipid metabolism-related gene expression in weaned pigs. Brit J Nutr..

[46] Shelton JL, Matthews JO, Southern LL, Higbie AD, Bidner TD, Fernandez JM (2004). Effect of nonwaxy and waxy sorghum on growth, carcass traits, and glucose and insulin kinetics of growing-finishing barrows and gilts. J Anim Sci.

[47] Deng JP, Wu X, Bin S, Li TJ, Huang RL, Liu Z (2010). Dietary amylose and amylopectin ratio and resistant starch content affects plasma glucose, lactic acid, hormone levels and protein synthesis in splanchnic tissues. J Anima Physio An N.

[48] Jeong J, Kwon E, Im S, Seo K, Baik M (2012). Expression of fat deposition and fat removal genes is associated with intramuscular fat content in longissimus dorsi muscle of Korean cattle steers. J Anim Sci.

[49] Munday M. Regulation of mammalian acetyl-CoA carboxylase: Portland Press Limited; 2002. 10.1042/bst0301059 12440972

[50] Chen J, Yang X-J, Tong H, Zhao R-Q (2004). Expressions of FAS and HSL mRNA in longissimus dorsi muscle and their relation to intramuscular fat contents in pig. J Agri Biotech.

[51] Tan B, Yin YL, Liu Z, Tang W, Xu H, Kong XF (2011). Dietary L-arginine supplementation differentially regulates expression of lipid-metabolic genes in porcine adipose tissue and skeletal muscle. J Nutr Biochem.

[52] Sobol M, Krawczyńska A, Skiba G, Raj S, Weremko D, Herman AP (2015). The effect of breed and feeding level on carcass composition, fatty acid profile and expression of genes encoding enzymes involved in fat metabolism in two muscles of pigs fed a diet enriched in n-3 fatty acids. A preliminary study. J Anim Feed Sci.

[53] Koonen DP, Glatz JF, Bonen A, Luiken JJ (2005). Long-chain fatty acid uptake and FAT/CD36 translocation in heart and skeletal muscle. BBA-Mol Cell Biol L.

[54] Klont R, Brocks L, Eikelenboom G (1998). Muscle fibre type and meat quality. Meat Sci.

[55] Kim GD, Jeong JY, Jung EY, Yang HS, Lim HT, Joo ST (2013). The influence of fiber size distribution of type IIB on carcass traits and meat quality in pigs. Meat Sci.

[56] Ryu Y, Kim BC (2005). The relationship between muscle fiber characteristics, postmortem metabolic rate, and meat quality of pig longissimus dorsi muscle. Meat Sci.

[57] Nakazato K, Tsutaki A (2012). Regulatory mechanisms of muscle fiber types and their possible interactions with external nutritional stimuli. J Phys Fit Sport Med.

