# Supplementary material for: Different dietary starch sources alter the carcass traits, meat quality, and the profile of muscle amino acid and fatty acid in finishing pigs
Source: J Anim Sci Biotechnol. 2020 Aug 7;11:78. doi: 10.1186/s40104-020-00484-9 (PMC7412799; doi:10.1186/s40104-020-00484-9)
Supplement: Supplementary file 1 — Additional file 1 Table S1. Primers used for host genes in this study. [file 40104_2020_484_MOESM1_ESM.docx]

**Additional files 1**

**Table S1.** Primers used for host genes in this study

| Gene^1^ | Primer sequence 5′-3′ | Accession no. | Size, bp |
| --- | --- | --- | --- |
| β-actin | Forward: CACGCCATCCTGCGTCTGGA  Reverse: AGCACCGTGTTGGCGTAGAG | DQ 845171 | 100 |
| *ACCα* | Forward: GGAGYATGTGGTTTAATTCGAAGCA | EF 618729 | 133 |
|  | Reverse: GTGGACCAGCTGACCTTGA |  |  |
| *PPARγ* | Forward: AAGACGGGGTCCTCATCTCC | NM 214379 | 149 |
|  | Reverse: CGCCAGGTCGCTGTCATCT |  |  |
| *LPL* | Forward: CTCGTGCTCAGATGCCCTAC | NM 214286 | 148 |
|  | Reverse: GGCAGGGTGAAAGGGATGTT |  |  |
| *FATP1* | Forward: GGAGTAGAGGGCAAAGCAGG | JN 713898 | 208 |
|  | Reverse: AGGTCTGGCGTGGGTCAAAG |  |  |
| *FAS* | Forward: AGCCTAACTCCTCGCTGCAAT | AY 183428 | 196 |
|  | Reverse: TCCTTGGAACCGTCTGTGTTC |  |  |
| *HSL* | Forward: GCAGCATCTTCTTCCGCACA | HM 591297 | 195 |
|  | Reverse: AGCCCTTGCGTAGAGTGACA |  |  |
| *CPT1B* | Forward: ATGGTGGGCGACTAACT | AY 181062 | 321 |
|  | Reverse: TGCCTGCTGTCTGTGAG |  |  |
| *SREBP* | Forward: GCGACGGTGCCTCTGGTAGT | AF 102873 | 218 |
|  | Reverse: CGCAAGACGGCGGATTTA |  |  |
| *MyHC-I* | Forward: CGTGGACTACAACATCATAGGC | NM 213855 | 152 |
|  | Reverse: CTTTGCCCTTCTCAACAGGT |  |  |
| *MyHC-IIa* | Forward: AAACCTCACGGAAGAGATGG | NM 214136 | 134 |
|  | Reverse: TCAGGGTGTTGACTTTGTCCT |  |  |
| *MyHC-IIx* | Forward: GAAACCGTCAAGGGTCTACG | NM 001104951 | 153 |
|  | Reverse: CGCTTCCTCAGCTTGTCTCT |  |  |
| *MyHC-IIb* | Forward: GATGTTCCTGTGGATGGTCA | NM 001123141 | 148 |
|  | Reverse: CTCGTTGGTGAAGTTGATGC |  |  |

^1^ *ACCα*, acetyl CoA carboxylase α; PPARγ, peroxisome proliferator-activated receptor γ; *LPL*, lipoprotein lipase; *FATP1*, fatty acid transport protein 1; *FAS*, fatty acid synthase; *HSL*, hormone-sensitive lipase; *CPT1B*, carnitine palmitoyl transferase 1B; *SREBP*, sterol regulatory element binding proteins; *MyHC*, myosin heavy chain.
